# Hepatitis B virus polymerase-specific T cell epitopes shift in a mouse model of chronic infection

**DOI:** 10.1186/s12985-021-01712-y

**Published:** 2021-12-07

**Authors:** Mohadeseh Hasanpourghadi, Mikhail Novikov, Dakota Newman, ZhiQuan Xiang, Xiang Yang Zhou, Colin Magowan, Hildegund C. J. Ertl

**Affiliations:** 1 Wistar Institute, 3601 Spruce Street, Philadelphia, PA 19104 USA; 2 Virion Therapeutics LLC, 7 Creek Bend Ct, Newark, DE 19711 USA

**Keywords:** Hepatitis B virus, Chronic hepatitis B infection, Vaccines, CD8^+^ T cell epitopes, Mouse model

## Abstract

**Background:**

Chronic hepatitis B virus (HBV) infection (CHB) is a significant public health problem that could benefit from treatment with immunomodulators. Here we describe a set of therapeutic HBV vaccines that target the internal viral proteins.

**Methods:**

Vaccines are delivered by chimpanzee adenovirus vectors (AdC) of serotype 6 (AdC6) and 7 (AdC7) used in prime only or prime-boost regimens. The HBV antigens are fused into an early T cell checkpoint inhibitor, herpes simplex virus (HSV) glycoprotein D (gD), which enhances and broadens vaccine-induced cluster of differentiation (CD8)^+^ T cell responses.

**Results:**

Our results show that the vaccines are immunogenic in mice. They induce potent CD8^+^ T cell responses that recognize multiple epitopes. CD8^+^ T cell responses increase after a boost, although the breadth remains similar. In mice, which carry high sustained loads of HBV particles due to a hepatic infection with an adeno-associated virus (AAV)8 vector expressing the 1.3HBV genome, CD8^+^ T cell responses to the vaccines are attenuated with a marked shift in the CD8^+^ T cells’ epitope recognition profile.

**Conclusions:**

Our data show that in different stains of mice including those that carry a human major histocompatibility complex (MHC) class I antigen HBV vaccines adjuvanted with a checkpoint inhibitor induce potent and broad HBV-specific CD8^+^ T cell responses and lower but still detectable CD4^+^ T cell responses. CD8^+^ T cell responses are reduced and their epitope specificity changes in mice that are chronically exposed to HBV antigens. Implications for the design of therapeutic HBV vaccines are discussed.

**Supplementary Information:**

The online version contains supplementary material available at 10.1186/s12985-021-01712-y.

## Introduction

More than 257 million humans worldwide suffer from chronic hepatitis B virus (CHB) infection [1], which can lead to liver cirrhosis and hepatocellular carcinoma (HCC), the 2^nd^ most common cause of cancer-related human deaths worldwide [2]. HBV-associated human mortality increased from 0.89 million to 1.45 million between 1990 and 2013 [3, 4] despite the availability of prophylactic HBV vaccines [5]. Antiviral drugs reduce HBV replication, but fail to eliminate the intranuclear viral genomes from infected hepatocytes and thereby do not cure [6–8].

During an acute infection CD8^+^ T cells can clear HBV-infected hepatocytes through cytolysis and the release of antiviral cytokines [9]. Persistent infections are associated with dysfunctional HBV-specific CD8^+^ T cell responses [10]. Due to sustained presence of antigens, induction of immunosuppressive cytokines [11], regulatory T cells (Tregs) [12], and the unique hepatic microenvironment [13], HBV-specific CD8^+^ T cells lose functions [14, 15]. Treatment with anti-Programmed Cell Death Protein 1. (PD1) antibodies may reverse CD8^+^ T cell dysfunction caused by exhaustion but has shown limited success in CHB patients [16]. The disappointing clinical outcome of checkpoint blockade in CHB patients contrasts results obtained in a pre-clinical woodchuck model of chronic hepadnaviral infection where a combination of anti-viral drugs, checkpoint blockade and an HBV-specific DNA vaccine led to sustained immunological control of the infection or complete viral clearance [17].

Here we describe pre-clinical results with a therapeutic vaccine to HBV, which induces potent and sustained CD8^+^ T cell responses that are relatively resistant to exhaustion and when combined with antiviral drugs may affect a functional cure of CHB. Implications of our findings for the development of a therapeutic HBV vaccine are discussed.

## Methods

### Cell lines

Human embryonic kidney (HEK) 293 cells and coxsackie adenovirus receptor (CAR)-transduced Chinese hamster ovary (CHO) cells were maintained in Dulbecco’s Modified Eagles medium (DMEM) supplemented with 10% fetal bovine serum (FBS) and antibiotics.

### Mice

Male 6 week-old C57BL/6, BALB/c and HLA-A2 transgenic (tg) (C57BL/6-*Mcph1*^*Tg(HLA−A2.1)1Enge*^/J) mice were purchased from Jackson Laboratory (Bar Harbor, ME, USA). Mice were housed at the Animal Facility of the Wistar Institute and treated according to approved protocols. Unless stated otherwise experiments were conducted with groups of 5 mice, 2 or 3 times.

### Production, purification, and titration of vectors

Early antigen (E)1 and partially E3-deleted AdC6 and AdC7 vectors expressing PolN, PolC or core within gD under the control of the early cytomegalovirus promoter were produced, purified and titrated as described previously [18]. They were formulated in 2.5% Glycerol/25 mM odium chloride (NaCl)/20 mM TRIS buffer, pH 8.0.

AAV8 vectors expressing the 1.3 genome of HBV genotype D were produced in HEK 293 cells by triple plasmid (p)Helper/pAAV-1.3HBV/pAAV8_capsid_ transfection using vectors at ratios of 1:0.5:0.5 as described [19]. HEK 293 cells were transfected with plasmids using polyethylenimine (PEI) at a 1:3 ratio of DNA: PEI. After a 72 h incubation in a 37 °C, 5% CO_2_ incubator cells were harvested by centrifugation and washed with phosphate buffered saline (PBS). The cell pellet was re-suspended in PBS and sonicated for 5 min with 1 min on and 1 min off at 50% amplitude to release the virus. Cell pellets were treated with 50U/mL of Benzonase, 0.5% sodium deoxycholate for 30 min in a 37 °C water bath. Cell debris was removed by centrifuging and the supernatant was subjected to centrifugation in a Beckman centrifuge with a 70.1 Ti rotor at 67,000 rpm for 2 h at 18 °C with maximum acceleration and no brake over an iodixanol step gradient (from bottom to top at 60%, 40%, 25%, and 15%). AAV particles were harvested from the 40–25% interface and dialyzed against PBS in a 100 kDa Millipore Ultra-50 unit. After dialysis, purified AAV was collected, aliquoted and stored at − 80 °C.

Aliquots of the purified AAV8-1.3HBV vectors were loaded on 10% sodium dodecyl sulfate (SDS) gel (Bio-Rad, Hercules, CA) and run for 1 h using 1 × running buffer. Silver staining was performed on the gel according to manufacturer’s instruction using Pierce™ Silver Stain Kit (ThermoScientific, Rockford, IL). Gels showed the expected 3 bands of AAV viral proteins 1–3 (not shown).

SYBR green quantitative (q) polymerase chain reaction (PCR) assays were performed with the SYBR Green Master Mix from ThermoFisher (Waltham, MA) to determine the AAV titers with insert-specific primers (Forward: TGAGAGGCCTGTATTTCCCTGC; Reverse: AACCCCGCCTGTAACACGAG). Briefly, 5µL aliquot of Iodixanol purified vector was treated with DNase I in PCR buffer at 37 °C for 30 min. Treatment samples were tenfold serially diluted in duplicates and amplified using the primers for PolN or PolC, and Sybr green master mix. Standard plasmids with known genome copies were included in each plate. Viral genome (vg) copies/mL were calculated based on cycle threshold values and the plasmid-derived standard curve.

### Protein expression

The Ad vectors were tested for protein expression upon transfection of HEK 293 cells or CHO cells stably transfected to express CAR. Briefly, 1 × 10^6^ cells/flask were infected for 48 h with ~ 1000 virus particles (vp)/cell of the AdC6 or AdC7 vectors expressing gDPoLN or gDPolC. Negative control cells were transfected with a characterized vector expressing gp140 of human immunodeficiency virus; uninfected cells served as an additional negative control. Cells were collected and lysed in radioimmunoprecipitation assay buffer (RIPA) buffer supplemented with a 1% protease inhibitor (Santa Cruz Biotechnology Inc., Dallas, TX). The lysate was stored at − 80 °C until further use. A 15 µl sample of protein was resolved on 12% SDS-polyacrylamid gel electrophoresis (NuPAGE™ 4-112 Bis–Tris Gel, Invitrogen, Carlsbad, CA) and transferred to a Immobilon-FL polyvinylidene difluoride (PVDF) membrane (Merck Millipore, Burlington, MA). The membrane was blocked in 5% powder milk overnight at 4 °C**.** The primary antibody to gD diluted to 1:1000 in saline (clone PA1-30233, Invitrogen, Carlsbad, CA) was added for 1 h at room temperature. Membranes were washed with 1X tris-buffered saline (TBS) with Polysorbate 20 (TBST) prior to incubating with horse radish peroxidase (HRP)-conjugated goat anti-rabbit secondary immunoglobulin (Ig)G (ab6721, Abcam, Cambridge UK) for 1 h at room temperature. Membranes were washed 3 times with 1X TBS-T. The developing agent Super Signal West Pico Chemiluminescent (Thermo Fisher Scientific, Waltham, MA) was added. Membranes were shaken in the dark for 5 min, dried, and developed.

### Vaccination and infection of mice

AdC6 or AdC7 vectors were diluted in sterile saline. A total volume of 200 µl containing the indicated numbers of vp was injected intramuscularly (i.m.) into the left hindlegs of mice. The AAV8-1.3HBV vector was diluted in sterile saline and injected at a volume of 300 µl into the tail vein.

### Preparation of peripheral blood mononuclear cells (PBMCs)

Mice were bled from the saphenous vein and blood was collected into 4% sodium carbonate and Liebowitz’s-15 (L-15) medium. PBMCs were purified by Ficoll® Paque Plus (GE Healthcare, Chicago, IL) gradient centrifugation for 30 min at 2800 rpm. Cells were washed and seeded into 96 roundbottom well plates (0.2–1 × 10^6^ cells per well).

### Collection of lymphocytes from livers and spleens

Spleens and livers were harvested from mice. Single cell suspension was generated by mincing spleens with mesh screens in L15 medium followed by passing cells through a 70 μm Falcon™ cell strainer (Thermo Fisher Scientific). Red blood cells (RBC) were lysed by 1X RBC lysis buffer (eBioscience, San Diego, CA). To obtain hepatic lymphocytes, livers were cut into small fragments and treated with 2 mg/ml Collagenase P, 1 mg/ml DNase I (all from Roche, Basel Switzerland) and 2% FBS (Tissue Culture Biologicals, Tulare, CA) in L15 under agitation for 1 h. Liver fragments were homogenized, filtrated through 70 μm strainers and lymphocytes were purified by Percoll-gradient centrifugation and washed with DMEM supplemented with 10% FBS.

### In vitro stimulation of lymphocytes

Lymphocytes were stimulated with various pools of peptides or individual peptides representing the HBV sequences present in the vaccines. Peptides were 15 amino acids in length and overlapped by 10 amino acids with the adjacent peptides. Individual peptides were diluted according to the manufacturer’s instructions in either water, DMSO, ammonia water, formic acid or N-methyl. For stimulation ~ 10^6^ lymphocytes plated in medium containing 2% FBS and BDGolgiplug Protein Transport Inhibitor (BD Bioscience; San Jose, CA), at 1.5 μl/ml were cultured with the peptide pools or individual peptides each present at a final concentration of 2 µg/ml for 5 h at 37 °C in a 5% CO_2_ incubator. Control cells were cultured without peptides.

### Intracellular cytokine staining (ICS) and analyses by flow cytometry

Following stimulation cells were incubated with anti-CD8- Allophycocyanin (APC) (clone 53-6.7, BioLegend, San Diego CA), anti-CD4-PerCp5 (clone Gk1.5, BioLegend), anti-CD44-Alexa Flour 700 (clone IM7, BioLegend) and Live/Dead™ fixable violet dye (Thermo Fisher Scientific) at + 4 °C for 30 min in the dark. Cells were washed once with PBS and then fixed and permeabilized with BD Cytofix/Cytoperm™ (BD Biosciences, San Jose, CA) for 20 min. Following fixation, cells were incubated with an anti-interferon (INF)-γ-FITC antibody (Clone, XMG1.2 BioLegend) at 4 °C for 30 min in the dark. Cells were washed and fixed in 1:3 dilution of BD Cytofix™ Fixation Buffer (BD Pharmingen, San Diego CA). They were analyzed by a BD fluorescent cell sorter (FACS) Celesta (BD Biosciences, San Jose, CA) and Data-Interpolating Variational Analysis (DiVa) software. Post-acquisition analyses were performed with FlowJo (TreeStar, Ashland, OR). Data shown in graph represents % of INF-γ production by CD8^+^ or CD44^+^CD8^+^ cells upon peptide stimulation. Background values obtained for the same cells cultured without peptide(s) were subtracted.

### Tetramer staining

Lymphocytes were stained with Live/Dead™ fixable violet dye (Thermo Fisher Scientific), anti-CD8-APC (clone 53-6.7, BioLegend) anti-CD44-Alexa Flour 700 (clone IM7, BioLegend), anti-EOMES-Alexa Fluor 488 (clone Dan11mag, eBioscience), anti-PD1-BV605 (clone 29F.1A12, BioLegend), anti-LAG3-BV650 (clone C9B7W, BioLegend), anti-T-bet-BV786 (clone 4B10, BioLegend), anti-CTLA4-PE-A (clone UC10-4B9, BioLegend), anti-TIM3-Pe-Cy7-A (clone RMT3-23, BioLegend) and an APC-labeled MHC class I tetramer (NIH tetramer Facility, Emory University, Atlanta GA) corresponding to amino acids 396-404 FAVPNLQSL (peptide 55) of the HBV polymerase at + 4 °C for 30 min in the dark. Cells were washed and analyzed by a BD FACS Celesta (BD Biosciences, San Jose, CA) and DiVa software. Post-acquisition analyses were performed with FlowJo (TreeStar, Ashland, OR).

### Titration of HBV genomes

Blood was harvested, sera were prepared, and DNA was extracted using DNeasy Blood & Tissue (Qiagen, Hilden, Germany) according to manufacturer’s protocol. The qPCR, which for each run contained serially diluted plasmids expressing the HBV sequence to provide a standard curve, was performed in a total volume of 20 µl including 1 µl of serum per PCR well. The following primers were used for amplification: forward primer: 5′-TGAGAGGCCTGTATTTCCCTGC-3′ and reverse primer 5′-AACCCCGCCTGTAACACGAG-3′. We used a ‘fast PCR’ with 40 cycles of 95 °C for 15 s followed by 60 °C for 1 min. Ct values of the standard curves were used to determine copy numbers per μl. Data were adjusted to 1 ml of serum. Water and sera from naïve mice served as controls.

### Statistics

For measured continuous variables (such as T cell frequencies), two group comparisons used t-tests, provided data were normally distributed. If the normality assumption was not valid, non-parametric Wilcoxon rank-sum tests were used. Multiple comparisons were analyzed by 2-way or 1-way Analysis of variance (ANOVA) as detailed in the Figure legends.

## Results

### Vaccine design and quality control

The polymerase and core amino acid (AA) sequences from HBV clades A, B, C, and D, which are genotypes that are commonly found in CHB patients [20], were downloaded as aligned ClustalW sequences from Hepatitis B Virus database (release version 45.0; updated on August 2, 2018) [21]. The AA sequences represents thousands of HBV genomes inputted from users across Europe (Additional file 1: Table S1). Consensus polymerase and core sequences were identified for each genotype using the Shannon Entropy tool (Los Alamos National Laboratory), which calculates the variation and frequency at each AA position. We selected for each consensus sequence two polymerase segments that are high in MHC class I binding epitopes for multiple HLA genotypes using the Immune Epitope Database (IEDB) analysis resource. (http://tools.iedb.org/main/tcell/). For AAs that varied between the different HBV genotypes, we used the IEDB software to select the AA with the lowest half maximal inhibitory concentration (IC_50_) of the corresponding peptide for multiple HLA class I alleles. We split the selected sequences into two inserts from the N or C terminal sequences, i.e., PolN and PolC. For core we used the entire sequence (Additional file 2: Table S2). Sequences were codon optimized and cloned into HSV gD after nucleotide 807 and then with the help of a transfer vector into the viral molecular clones of AdC6 and AdC7. Once vectors, called AdC6-gDPolN, AdC7-gDPolN, AdC6-gDPolC, AdC7-gDPolN, AdC6-gDCore and AdC7-gDCore had been rescued, quality controlled and titrated, protein expression was confirmed in CHO cells that had been stably transduced with CAR by Western blots using a gD-specific antibody (Additional file 3: Fig. S1). We decided to not include sequences from the HBV surface antigen (HBsAg) into our vaccine. HBsAg highly variable between isolates and is present at very high levels in CHB patients, which most likely results in terminal exhaustion of specific T cells rendering them unresponsive to a vaccine.

### T cell responses to the AdC vectors

**Fig. 1:**
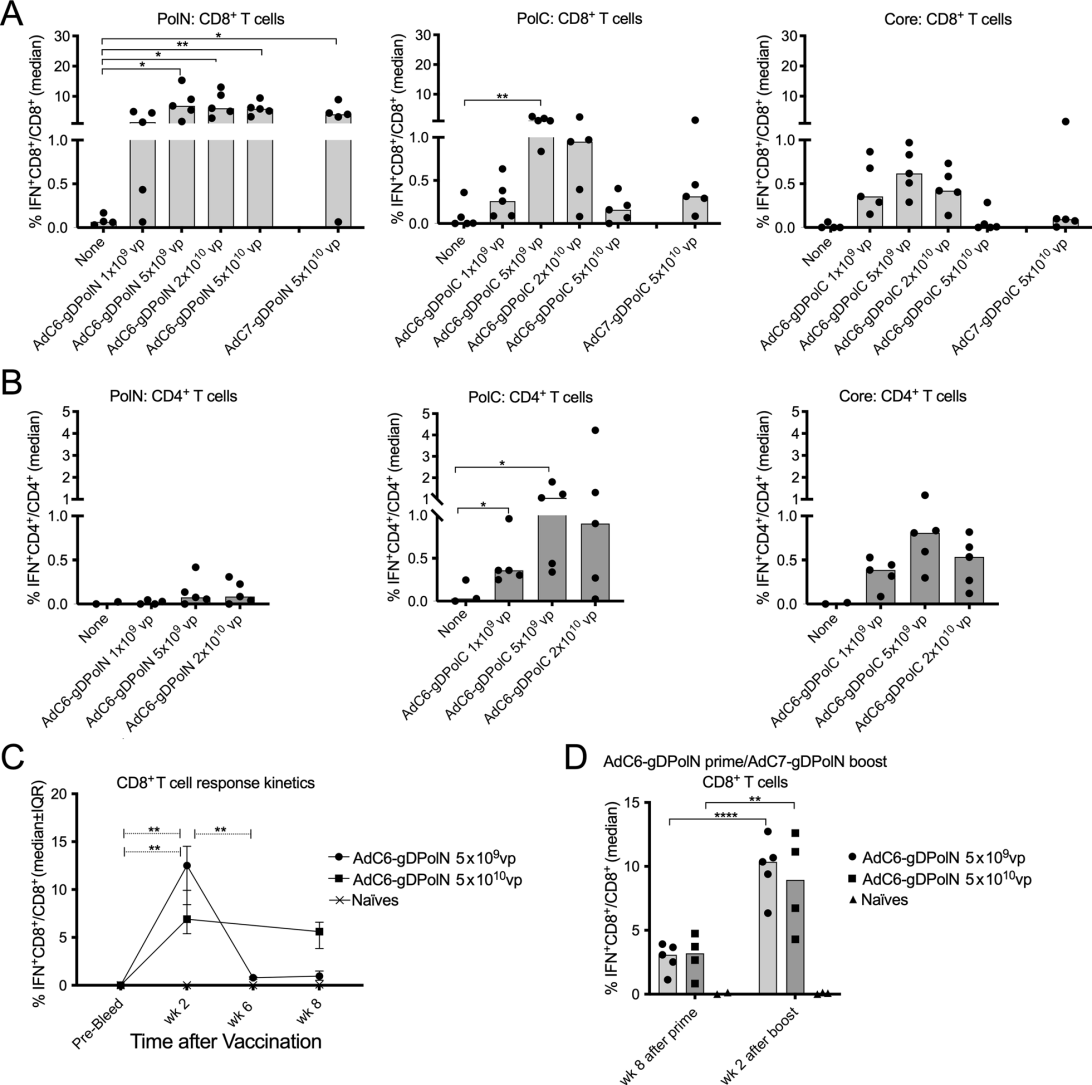
T cell responses to AdC-gDPolN, AdC-gDPolC and AdC-gDCore. Male C57Bl/6 mice (n = 5) were injected with the indicated doses of the AdC6-gDPolC, AdC7-gDPolC, AdC6-gDPolN, AdC7-gDPolN, AdC6-gDCore or AdC7-gDCore vectors. Naïve mice served as controls. **a**, **b** Fourteen days later PMBCs were tested by ICS for CD8^+^ and CD4^+^ T cells producing IFN-γ in response to peptide pools reflecting the HBV inserts. Data are shown for individual mice with lines indicating medians. Lines with stars above indicate significant differences by 2-way Anova. (*) p ≤ 0.05–0.01, (**) p ≤ 0.01–0.001, (***) p ≤ 0.001–0.0001, (****) p ≤ 0.0001. **c** Mice were immunized with the indicated doses of AdC6-gDPolN. PBMCs were tested at different times after vaccination by ICS. Naïve mice served as controls. Graph shows medians ± IQR. Lines with stars above indicate significant differences within one vaccine group tested at different times by one-way ANOVA. **d** Groups of C57Bl/6 mice were primed with the indicated doses of AdC6-gDPolN. PBMCs were tested 8 weeks later. The next day mice were boosted with the same doses of the AdC7-gDPolN vector. PBMCs were tested 2 weeks later. Graph shows frequencies of IFN-γ producing CD8^+^ T cells from individual mice. Lines with stars above indicate significant differences by two-way ANOVA

AdC6 vectors expressing gDPolN, gDPolC or gDCore were injected i.m. at 1 × 10^9^, 5 × 10^9^, 2 × 10^10^, and 5 × 10^10^ vp into C57Bl/6 mice. AdC7 vectors expressing the same inserts were injected at 5 × 10^10^vp. Naïve mice served as controls. Fourteen days later, when T cell responses to Ad vector inserts generally peak, mice were bled and PBMCs were stimulated with pools of peptides representing the sequences of the HBV inserts. Cells were tested by ICS upon treatment with fluorochrome-labeled antibodies against surface markers and intracellular IFN-γ and analyzed by flow cytometry. The AdC6-gDPolN and AdC7-gDPolN vaccines induced potent CD8^+^ T cell responses (Fig. 1A); CD4^+^ T cell responses only tested for the AdC6 vectors were marginal (Fig. 1B). The gDPolC expressing vaccines induced lower CD8^+^ but higher CD4^+^ T cell responses (Fig. 1A and B). T cell responses to core were detectable but comparatively low (Fig. 1A and B). To determine CD8^+^ T cell response kinetics we immunized mice with 5 × 10^9^ or 5 × 10^10^vp of the AdC6-gDPolN vector. Responses to the low vector dose were high 14 days after vaccination, contracted by week 6 and remained stable thereafter. Responses to the high vector dose were sustained over the observation period (Fig. 1C). To determine if CD8^+^ T cell responses could be increased further by a boost with a heterologous Ad vector, mice were primed with 5 × 10^9^ or 5 × 10^10^ vp of the AdC6-gDPolN vector. They were boosted 8 weeks later, when responses had stabilized, with the same doses of the AdC7-gDPolN vector. CD8^+^ T cell responses were tested from blood just before and 2 weeks after the boost. As shown in Fig. 1D, regardless of the vaccine dose the boost caused a significant increase in frequencies of PolN-specific CD8^+^ T cells.

**Fig. 2:**
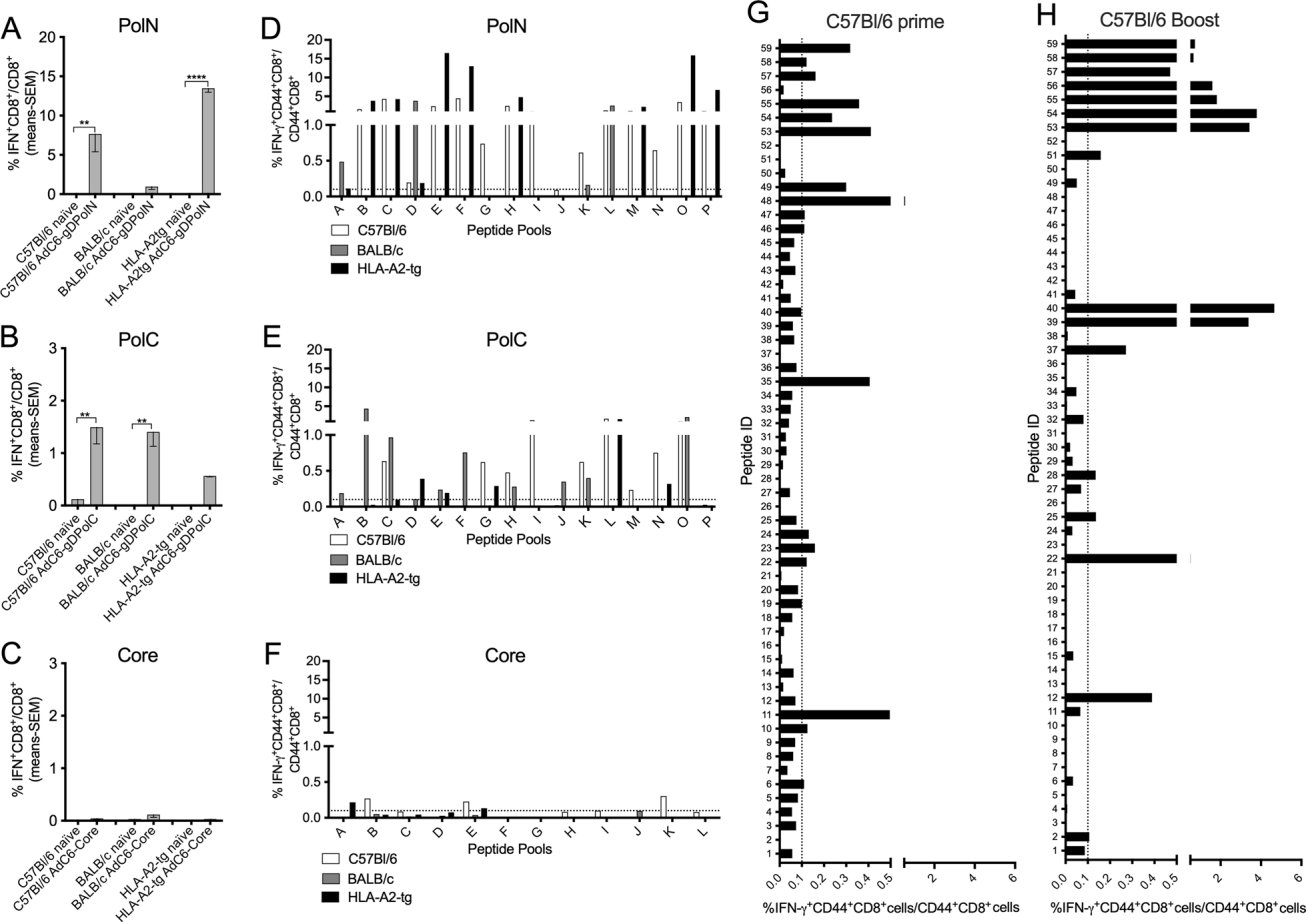
CD8^+^ T cell responses in different mouse strains. **a**–**f** Mice (n = 5) were injected with of 5 × 10^10^ vp (C57Bl/6 and BALB/c mice) or 5 × 10^9 ^vp (HLA-A2 tg mice) of AdC6-gDPolN [**a**] AdC6-gDPolC [**b**] or AdC6-gDCore [**c**]. PBMCs were tested 2 (AdC6-gDPolN, AdC6-gDPolC) or 6 (AdC6-gDCore) weeks later by ICS for frequencies of HBV-specific CD8^+^ T cells. Naïve mice served as control. Lines with stars above indicate significant differences by t-tests. **d**–**f** Splenocytes of mice were tested ~ 2 months after immunization upon stimulation with peptide pools. Splenocytes were pooled from 5 mice and data are shown as frequencies over CD44^+^CD8^+^ cells. The dotted line at 0.1% reflects the experimental cut-off for positive samples. CD44^+^CD8^+^ cells from naïve mice tested in parallel were negative. **G** Mice were primed with 5 × 10^10^vp of AdC6-gDPolN. **h** Mice were prime with 5 × 10^10^vp of AdC6-gDPolN and then boosted 8 weeks later with 5 × 10^10^vp of AdC7-gDPolN. **g**, **h** Pooled splenocyts were tested 6 weeks after completion of vaccination by ICS for IFN-γ production in response to individual peptides representing the PolN sequence. Data are shown as frequencies over CD44^+^CD8^+^ cells. The dotted line at 0.1% reflects the experimental cut-off for positive samples. CD44^+^CD8^+^ cells from naïve mice tested in parallel were negative

To assess responses in different mouse strains, including mice expressing a common human leukocyte antigen (HLA), AdC6-gDPolN, AdC6-gDPolC or AdC6-gDCore vectors were injected into C57Bl/6, BALB/c and HLA-A2-tg mice at doses of 5 × 10^10^vp (C57Bl/6 and BALB/c mice) or 5 × 10^9^vp (HLA-A2-tg mice). PBMCs were tested 2 (AdC6-gDPolN and AdC6-gDPolC) or 6 weeks (AdC6-gDCore) later for frequencies of HBV-specific, IFN-γ-producing CD8^+^ T cells. The Ad-gDPolN vector resulted in very robust responses in C57Bl/6 and HLA-A2-tg mice. BALB/c mice showed lower responses (Fig. 2A). The PolC sequence was less immunogenic in HLA-A2-tg mice but elicited higher responses in BALB/c mice (Fig. 2B). CD8^+^ T cell responses to core, when tested later at 6 weeks after immunization remained low (Fig. 2C).

### Breadth of CD8^+^ T cell responses

Viruses commonly mutate and thereby escape immune surveillance. This can be prevented by vaccines that induce responses to multiple epitopes. To determine the breadth of vaccine-induced HBV-specific CD8^+^ T cells, we first tested pooled splenocytes from mice that had been immunized as described above 2 months before using peptides that were arranged into pools of 8 peptides each so that each peptide was uniquely present in 2 pools (Additional file 2: Table S2). We tested T cells 2 months after immunization rather than early at 2 weeks when T cell frequencies peak to allow for further maturation of the T cell specificity profile that as we showed previously changes over time [19]. For the two polymerase fragments we used 16 pools, for core we used 12 pools. CD8^+^ T cells from C57Bl/6 mice recognized 14 PolN peptide pools, BALB/c mice responded to 4 pools while HLA-A2 tg mice responded to 10 pools (Fig. 2D). AdC6-gDPolC-immune C57Bl/6 mice responded to 8 pools, BALB/c mice responded to 9 pools while HLA-A2-tg mice scored positive on 5 pools (Fig. 2E). Within the core sequence CD8^+^ T cells from C57Bl/6 mice recognized 3 peptide pools, BALB/c mice did not score positive on any of the peptide pools while HLA-A2-tg mice responded to two pools (Fig. 2F).

We repeated the experiment for C57Bl/6 mice primed with the 5 × 10^10^vp of AdC6-gDPolN or first primed with 5 × 10^10^vp of AdC6-gDPolN and then boosted with 5 × 10^10^ vp of AdC7-gDPolN. Splenocytes were stimulated 6 weeks after the prime or the boost with individual peptides representing the PolN sequence. CD8^+^ T cells from mice that had been primed only responded to 17 out of 54 peptides (Fig. 2G) while those from mice that received a boost responded to 16 out of 54 peptides (Fig. 2H). The boost tended to increase responses to peptides that scored positive in both groups. Some peptides were only recognized after the prime while others only scored positive after the boost. Overall, although it increased CD8^+^ T cell responses, the boost failed to augment their breadth.

### HBV animal model

**Fig. 3:**
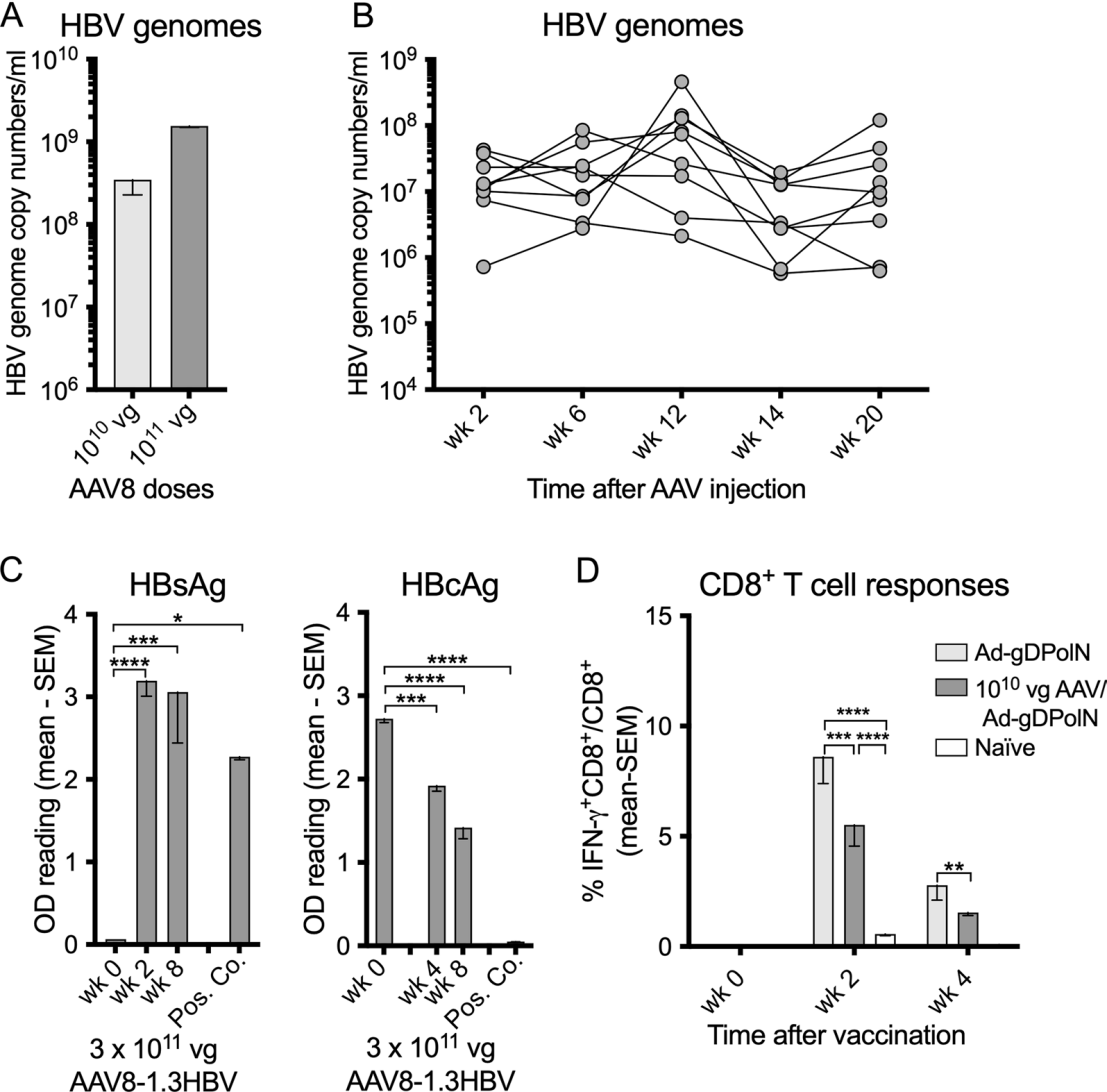
CHB model. **a** C57Bl/6 mice (n = 3) were injected with 10^10^ or 10^11^vg of the AAV8-1.3HBV vector. Sera collected at 2 weeks after injection were screened for HBV genome copies. **b** The experiment was repeated with 10^10^vg of AAV8-1.3HBV and sera from 9 mice were tested for 20 weeks. Data for different PCR runs were normalized to the first set of data obtained with week 2 sera, which were tested in each PCR run. **c** Mice (n = 5) were injected with 3 × 10^11^ vg of AAV8-1.3HBV. Sera were harvested at baseline and at different times after injection and tested by a direct ELISA for HBsAg (left graph) or a competition ELISA for HBcAg (right graph). Sera from naïve mice serves as negative controls. Samples provided by the kits served as positive controls. Lines with stars above indicate significant differences by one-way ANOVA. **d** C57Bl/6 mice (n = 5) were injected with 10^10^vg of AAV8-1.3HBV vector. Together with naïve mice they were vaccinated 4 weeks later with 5 × 10^9^vp of the AdC6-gDPolN vector. At the indicated times after vaccination mice were bled and PBMCs were tested for PolN-specific CD8^+^ T cells. PBMCs from naïve mice served as controls. Lines with stars above indicate significant differences by two-way ANOVA

Neither humans, chimpanzees nor tupaias, the natural hosts for HBV, are suited for pre-clinical assessment of vaccine-induced T cell responses. We therefore developed a mouse model based on an AAV8-1.3HBV vector [22]. Male C57Bl/6 (n = 3) mice were injected intravenously (i.v.) with 10^10^ or 10^11^ vg of the AAV8-1.3HBV vector and then tested 2 weeks later for circulating HBV genome copy numbers. Mice developed robust levels of circulating HBV genomes (Fig. 3A). The experiment was repeated in male C57Bl/6 mice (n = 9) in which the 10^10^ vg dose of the AAV8-1.3HBV vector was shown to result in sustained levels of circulating HBV genome copy numbers (Fig. 3B). Viral proteins were detected in sera of mice (n = 5) that for this experiment had been injected with 3 × 10^11^ vg of the AAV8-1.3HBV vector (Fig. 3C). Levels of the hepatitis B virus surface antigen (HBsAg) were high presumably reflecting production of excess protein as is typical for HBV [23]. Levels of hepatitis core antigen (HBcAg) were modest. PBMCs from mice that had been injected with the AAV vectors were tested for a period of 8 weeks for HBV PolN-specific CD8^+^ T cell responses; they were below detection (not shown).

### Vaccine induced CD8^+^ T cells in AAV8-1.3HBV infected mice

We tested the AdC6-gDPolN vaccine for induction of CD8^+^ T cells to the HBV insert in AAV8-1.3HBV vector carrying mice. C57Bl/6 mice were injected i.v. with 10^10^ vg of the AAV8-1.3HBV vector; 4 weeks later they were vaccinated with 5 × 10^9^vp of the AdC6-gDPolN vector. Other mice received vaccine only. Naïve mice served as controls. PBMCs were harvested at baseline and then 2 and 4 weeks after vaccination and frequencies of insert-specific IFN-γ-producing CD8^+^ T cells were determined. The magnitude of vaccine-induced CD8^+^ T cell responses was attenuated in mice that chronically expressed HBV antigens (Fig. 3D).

### T cell response breadth in the CHB mouse model

**Fig. 4:**
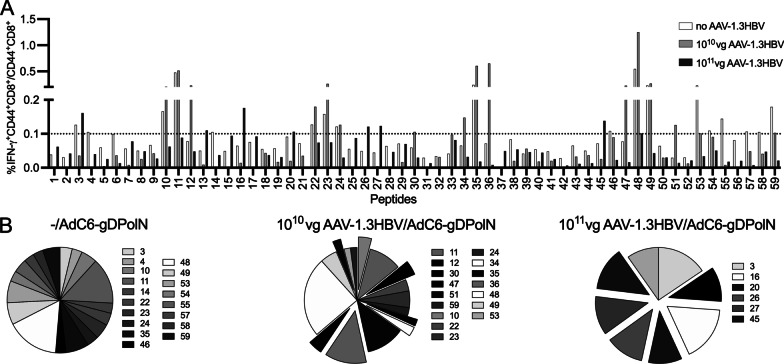
The effect of AAV8-1.3HBV on the breadth of AdC6-gDPolN-induced CD8^+^ T cell responses. **a** C57Bl/6 mice (n = 5) were injected with no, 10^10^ or 10^11^vg of AAV8-1.3HBV vector. They were vaccinated 4 weeks later with 5 × 10^10^vp of the AdC6-gDPolN vaccine. Eight weeks later CD44^+^CD8^+^ T cells from spleens were probed for IFN-γ production in response to the individual peptides of the PolN sequence. The line at 0.1 reflects the cut-off for positive samples. **b** shows the relative distribution of responses to individual peptides to the total response. The pop-out slices highlight peptides that were not recognized by CD8^+^ T cells from mice that only received the vaccine. Legends indicate the peptides that scored positive

The next set of experiments was conducted to evaluate if presence of HBV antigens influences the breadth of vaccine-induced CD8^+^ T cells, by selectively impairing CD8^+^ T cells to selected epitopes. Mice were first injected with 10^10^ or 10^11^ vg of the AAV-1.3HBV vector. Four weeks later these mice as well as a control group were vaccinated with 5 × 10^10^vp AdC6-gDPolN. Splenocytes were tested 8 weeks after vaccination for CD8^+^ T cell responses to the individual peptides of the PolN panel. Vaccinated control mice responded to 18/59 peptides (Fig. 4A and B). Pre-exposure to 10^10^ vg of AAV8-1.3HBV reduced responsiveness to 16 peptides with a loss in responses to some of the N-terminal peptides (Fig. 4A and B). Mice that had been pre-treated with 10^11^vg of the AAV8-1.3HBV vector responded to 8 peptides most of which had not triggered a response in the control vaccine group. These results suggest that chronic exposure to antigen not only reduces vaccine-induced CD8^+^ T cell responses but also shifts their specificity profile.

### CD8^+^ T cell responses in liver

**Fig. 5:**
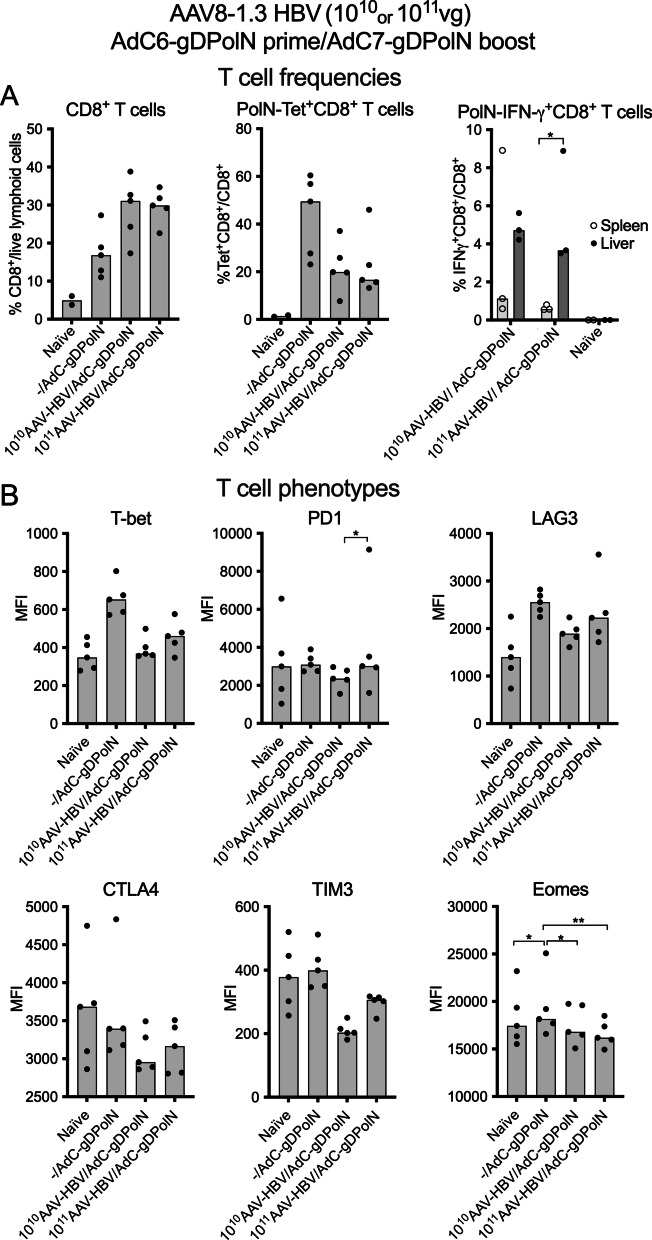
Phenotypes and Functions of Hepatic PolN-Specific CD8^+^ T Cells. **a** C57Bl/6 mice (n = 5) were injected with nothing, 10^10^ or 10^11^vg of AAV8-1.3HBV. They were vaccinated 4 weeks later with 5 × 10^9^ vp of AdC6PolN vector. Lymphocytes were isolated from individual livers 8 weeks later. Liver lymphocytes from naive mice served as controls. The left graph shows frequencies of CD8^+^ T cells over all live single lymphoid cells. The middle graph shows frequencies of tetramer^+^CD8^+^ T cells over all CD8^+^ T cells. The left graph shows frequencies of CD8^+^ T cells producing IFN-γ in response to the PolN peptide pool. Lines with stars above indicate significant differences by multiple t-test. **b** Cells were stained with antibodies to the indicated markers and gated on either CD44^-^tetramer^-^CD8^+^ T cells (naïve) or tetramer^+^CD8^+^ T cells. Graphs show the mean fluorescent intensity of the dyes linked to antibodies to the indicated markers. Lines with stars above indicate significant differences by Tukey's multiple comparisons test

We assessed vaccine-induced CD8^+^ T cells in livers of normal or AAV8-1.3HBV-infected mice to determine if the reduction of responses in blood in presence of high loads of HBV particles was mirrored in livers. C57Bl/6 mice were injected with 10^10^ or 10^11^vg of AAV8-1.3HBV. They were vaccinated 4 weeks later with 5 × 10^9^vp of the AdC6PolN vector followed 8 weeks later by a boost with the AdC7-gDPolN vector given at the same dose. Cells were isolated from livers 8 weeks later, when in chronic infection models T cells have undergone differentiation towards exhaustion [24] and stained with a live cells stain, an antibody to CD8 and a tetramer for an immunodominant MHC class I epitope within the PolN sequence. CD8^+^ T cells from livers of mice injected with the AAV8-1.3HBV vector showed in comparison to those that had only been vaccinated increases in CD8^+^ T cells. Nevertheless, percentages of PolN-specific tetramer^+^CD8^+^ T cells were markedly lower (Fig. 5A). This reduction was not merely a reflection of increased recruitment of CD8^+^ T cells with unrelated specificities as numbers of tetramer positive CD8^+^ T cells were also reduced in AAV8-1.3HBV infected mice (not shown). As tetramer staining fails to give insight into T cell functionality, we isolated lymphocytes from mice that after injection of 10^10^ or 10^11^ vg of AAV8-1.3HBV had been vaccinated with 5 × 10^10^ vp of AdC6-gDPolN followed by a boost with 5 × 10^10^ vp of AdC7-gDPolN. CD8^+^ T cells from spleens and liver were tested two months after the boost for production of IFN-γ in response to the PolN-specific peptide pool. As shown in Fig. 5A, cytokine-producing CD8^+^ T cells could be detected in livers and spleens. At the higher AAV8-1.3HBV dose frequencies of hepatic PolN-specific IFN-γ^+^CD8^+^ exceeded those in spleen. To test if exhaustion contributed to the loss of vaccine-induced CD8^+^ T cells we determined expression levels of T-box transcription factor (T-bet), a transcription factor that controls T cell functions and the exhaustion markers PD1, lymphocyte activating gene 3 (LAG3), cytotoxic T lymphocyte associated protein 4 (CTLA4) and Eomesodermin (Eomes) by PolN-specific CD8^+^ T cells from AdC6-gDPolN-vaccinated mice that had or had not been pre-treated with 10^10^ or 10^11^ vg of the AAV8-1.3HBV vector (Fig. 5B).

Mice with high loads of HBV particles showed declines in the expression of T-bet, which is compatible with the observed loss in frequencies of PolN-specific IFN-γ-producing CD8^+^ T cells in blood. Other exhaustion markers, i.e., PD1, LAG3, CTLA4, and Eomes, tended to be higher on tetramer^+^CD8^+^ T cells than on naïve CD8^+^ T cells but showed no significant differences between the experimental groups. There were significant differences for T-cell immunoglobulin domain and mucin domain 3 (TIM3), which showed a dose-dependent decline in AAV8-1.3HBV pretreated mice. Overall, these data argue against CD8^+^ T cell exhaustion in the mouse CHB model.

## Discussion

Most humans are able to clear an acute HBV infection, but some develop CHB. Available drugs reduce HBV replication and lower viral loads and the associated liver damage. Drugs have to be given for extended periods of time as none affects a cure and virus may resurge upon treatment discontinuation. Immunomodulators offer alternative therapies. During an acute infection innate response cytokines including type 1 IFNs, tumor necrosis factor (TNF)-α and the apolipoprotein B mRNA editing catalytic polypeptide-like (APOBEC) pathway can suppress virus replication [25, 26]. Innate responses, to which HBV has evolved numerous escape mechanisms [27], facilitate induction of adaptive immune responses and of those HBV-specific CD8^+^ T cells can control the virus and eliminate virus-infected cells [28, 29].

During CHV HBV-specific CD8^+^ T cells lose functions [30, 31]. HBV-specific CD8^+^ T cell responses can be restored at least temporarily by adoptive transfer of T cells that are genetically modified to express a receptor against an HBV antigen. Preclinical models have yielded promising data with HBV-specific chimeric antigen receptor T cells [32], nevertheless, due to the complexity of generating such T cells, they would not be widely available. Others have explored the use of checkpoint inhibitors to rescue exhausted HBV-specific CD8^+^ T cells. Terminally exhausted CD8^+^ T cells that express multiple immunoinhibitory surface markers and have undergone epigenetic changes cannot be rescued by checkpoint blockade, which mainly targets progenitor exhausted T cells [33], which would eventually undergo transcriptional changes and differentiate into terminally exhausted T cells [34]. In clinical trials treatment of HCC patients with a monoclonal antibody to PD1 or CTLA4 showed some, albeit limited, clinical benefits [16, 35]. Considering that checkpoint inhibitors commonly cause serious adverse events this treatment may not be appropriate in otherwise healthy CHB patients. Vaccines that aim to induce HBV-specific CD8^+^ T cells have shown efficacy in pre-clinical models especially if they were given together with an anti-PDL1 antibody [17] but overall yielded disappointing results in clinical trials even when they were combined with anti-viral treatment or checkpoint inhibitors [16, 36, 37].

Here we describe the immunogenicity of HBV vaccines that combine core or segments of HBV polymerase with an inhibitor of an early immune checkpoint in a pre-clinical mouse model. As we reported earlier, inhibition of the BTLA-HVEM checkpoint by HSV gD at the time of T cell activation enhances and broadens CD8^+^ T cell responses [38, 39]. gD specifically promotes CD8^+^ T cells that recognise subdominant epitopes and these T cells are less prone to become impaired during chronic antigen exposure [39]. As gD is encoded by the vaccine and largely remains localized at the site of injection and draining lymph nodes, adverse events beyond those typically observed after vaccination with an Ad vector [40] are unlikely. Our results show that the vaccines, especially those expressing the N-terminus of polymerase, induce potent and sustained CD8^+^ T cell responses to multiple epitopes that can be boosted by a heterologous AdC vector. In our current study, mice with high levels of HBV upon injection of the AAV8-1.3HBV vector generate lower frequencies of functional HBV-specific CD8^+^ T cells upon vaccination and in addition exhibit a shift in the CD8^+^ T cells’ epitope profile, as has been observed by others in chronic infection mouse models [41, 42]. This raises the question if therapeutic HBV vaccines should focus on inserts that induce more modest T cell responses to subdominant epitopes, rather than inserts that are plentiful in dominant epitopes; the latter may induce more potent CD8^+^ T cell responses in HBV-naïve individuals. On the other hand, vaccines rich in subdominant epitopes may fare better in patients with CHB as they may induce HBV-specific naïve CD8^+^ T cells that had not been stimulated by the virus and that had therefore not been subjected to the immunosuppressive conditions of a chronic virus infection.

The AAV8-1.3HBV model is a surrogate for CHB that does not completely mirror the complexity of this disease in humans. The AAV8-1.3HBV vector, which persists in the liver of mice, does not cause any overt liver pathology and results if given at doses ≥ 10^11^ vg in a few foci of infiltrating lymphocytes (data not shown). Mice injected i.v. with AAV8-1.3HBV did not develop detectable CD8^+^ T cell responses to polymerase and although the vector affected the magnitude of CD8^+^ T cell responses to subsequent vaccination, there was limited evidence that T cells were driven towards exhaustion; levels of exhaustion markers, including Eomes, which has recently been linked to exhaustion [43], were not elevated on or in vaccine-induced CD8^+^ T cells in AAV8-1.3HBV treated mice. The only marker suggestive of T cell dysfunctions was T-bet, which slightly decreased in the presence of HBV. The early reduction in vaccine-insert-specific CD8^+^ T cell frequencies is suggestive of loss of activation rather than antigen-driven CD8^+^ T cell exhaustion and remains to be investigated in more depth.

AAV vectors given to liver have been shown previously to induce transgene product-specific B and T cell tolerance due to stimulation of Tregs [44, 45]. We assume that the reduced CD8^+^ T cell responses to the Ad-gDPolN vaccine in mice that were pre-treated with the AAV8-1.3HBV vector may reflect that actions of Tregs. This is also compatible with our finding of the preferential loss of stimulation of CD8^+^ T cells to immunodominant epitopes within the vaccine insert as previous work has shown them to be particularly susceptible to Treg-mediated inhibition [46].

## Conclusion

In summary, our data show that in different stains of mice including those that carry a human MHC class I antigen the HBV vaccines induce potent HBV-specific CD8^+^ T cell responses and lower but still detectable CD4^+^ T cell responses. CD8^+^ T cell responses are exceptionally broad, which should prevent viral escape through mutations. CD8^+^ T cell responses are attenuated in mice with high loads of HBV particles although high frequencies of vaccine-induce functional HBV-specific CD8^+^ T cells remain detectable at the site of HBV infection. Chronic exposure to HBV antigens causes a shift in the specificity of vaccine-induced CD8^+^ T cells with a preferential loss of those directed to immunodominant epitopes. This in turn suggests that therapeutic vaccines should express sequences that are able to boost the remaining responses rather than target those that are lost during chronic infections.

## Supplementary Information

**Additional file 1. Table S1**. The polymerase amino acid and core sequences from HBV clades A, B, C, and D

**Additional file 2. Table S2**. Consensus polymerase and core sequences

**Additional file 3. Figure S1**. Expression of the gD fusion proteins by the vaccines. The graphs show results of Western blot for CHO-CAR cells infected with 10^3^vp/cells of the AdC6-gDPolC and AdC6-gDPolN vectors or HEK293 cells infected with the same doses of the AdC6-gDCore, AdC7-gDPolC, AdC7-gDPolN or AdC7-gDCore vectors. An AdC vector expressing gp140 of HIV-1 was used as a negative control. An AdC vector expressing a gD-HPV oncoprotein fusion protein was used as a positive control. The blots were probed with an antibody to gD, stripped, and then tested with an antibody to ß-actin as a loading control (not shown)

## Data Availability

Data and materials are available on request.

## Abbreviations

AAV: Adeno-associated virus
AdC: Chimpanzee adenovirus vectors
AdC6: Chimpanzee adenovirus vectors of serotype 6
AdC7: Chimpanzee adenovirus vectors of serotype 7
APC: Allophycocyanin
APOBEC: Apolipoprotein B mRNA editing catalytic polypeptide-like
ANOVA: Analysis of variance
CAR: Coxsackie adenovirus receptor
CD: Cluster of differentiation
CHB: Chronic hepatitis B
CTLA4: Cytotoxic T-lymphocyte associated protein 4
DiVa: Data-interpolating variational analysis
DMEM: Dulbecco's modified eagle medium
DMSO: Dimethyl sulfoxide
E: Early antigen
Eomes: Eomesodermin
FACS: Fluorescent cell sorter
FBS: Fetal bovine serum
gD: Glycoprotein D
HBV: Hepatitis B virus
HCC: Hepatocellular carcinoma
HEK: Human embryonic kidney cells
HLA: Human leukocyte antigen
HRP: Horse radish peroxidase
HSV: Herpes simplex virus
IC_50_: Half maximal inhibitory concentration
ICS: Intracellular cytokine staining
IEDB: Immune Epitope Database
IFN: Interferon
Ig: Immunoglobulin
L-15: Liebowitz’s-15 medium
LAG3: Lymphocyte activating 3
MHC: Major histocompatibility complex
mRNA: Messenger RNA
NaCl: Sodium chloride
p: Plasmid
PAGE: Polyacrylamide gel electrophoresis
PBMCs: Peripheral blood mononuclear cells
PBS: Phosphate buffered saline
PCR: Polymerase chain reaction
PD1: Programmed death protein 1
PEI: Polyethylenimine
Pol: DNA polymerase
PolC: C-terminus polymerase
PolN: N-terminus polymerase
PVDF: Polyvinylidene difluoride
q: Quantitative
RBC: Red blood cell
RIPA: Radioimmunoprecipitation assay
SDS: Sodium dodecyl sulfate
T-bet: T-box transcription
TBS: Tris-buffered saline
TBST: TBS with polysorbate 20
tg: Transgenic
TIM3: T-cell immunoglobulin domain and mucin domain 3
TNF: Tumor necrosis factor
Tregs: Regulatory T cells
vg: Viral genome copies
vp: Virus particles
