# Supplementary material for: Hepatitis B virus polymerase-specific T cell epitopes shift in a mouse model of chronic infection
Source: Virol J. 2021 Dec 7;18:242. doi: 10.1186/s12985-021-01712-y (PMC8650432; doi:10.1186/s12985-021-01712-y)
Supplement: Supplementary file 1 — Additional file 1. Table S1. The polymerase amino acid and core sequences from HBV clades A, B, C, and D [file 12985_2021_1712_MOESM1_ESM.pdf]

Additional Information Table 1. Consensus sequences for HBV polymerase and core

The table shows the consensus sequences for genotypes A-D and the consensus sequence for all 4 genotypes for polymerase and core. Data are based on: https://www.hiv.lanl.gov/cgi- bin/ENTROPY/entropy_main.cgi.

Polymerase							
Position	Consensus	Pol A aa consensus	Pol B aa consensus	Pol C aa consensus	Pol D aa consensus	PolN sequence	PolC sequence
Analyzed Genomes (no)		1,482	2,800	2,768	1,579		
1	M	M	M	M	M		
2	P	P	P	P	P	P	
3	L	L	L	L	L	L	
4	S	S	S	S	S	S	
5	Y	Y	Y	Y	Y	Y	
6	Q	Q	Q	Q	Q	Q	
7	H	H	H	H	H	H	
8	F	F	F	F	F	F	
9	R	R	R	R	R	R	
10	NoCon	K	K	K	R	K	
11	L	L	L	L	L	L	
12	L	L	L	L	L	L	
13	L	L	L	L	L	L	
14	L	L	L	L	L	L	
15	D	D	D	D	D	D	
16	NoCon	D	-	D	D	E	
17	NoCon	G	-	-			
18	NoCon	T		-			
19	NoCon	-					
20	E	E	E	E	E	E	
21	A	A	A	A	A	A	
22	G	G	G	G	G	G	
23	P	P	P	P	P	P	
24	L	L	L	L	L	L	
25	E	E	E	E	E	E	
26	E	E	E	E	E	E	
27	E	E	E	E	E	E	
28	L	L	L	L	L	L	
29	P	P	P	P	P	P	

Core						
Position	Consensus	Cor A aa consensus	Cor B aa consensus	Cor C aa consensus	Cor D aa consensus	Core sequence
Analyzed Genomes (no)		1,482	2,800	2,768	1,579	
1	M	M	M	M	M	
2	D	D	D	D	D	D
3	I	I	I	I	I	I
4	D	D	D	D	D	D
5	P	P	P	P	P	P
6	Y	Y	Y	Y	Y	Y
7	K	K	K	K	K	K
8	E	E	E	E	E	E
9	F	F	F	F	F	F
10	G	G	G	G	G	G
11	A	A	A	A	A	A
12	NoCon	T	S	S	T	T
13	V	V	V	V	V	V
14	E	E	E	E	E	E
15	L	L	L	L	L	L
16	L	L	L	L	L	L
17	S	S	S	S	S	S
			-			
			-	-		
18	F	F	F	F	F	F
19	L	L	L	L	L	L
20	P	P	P	P	P	P
21	S	S	S	S	S	S
22	D	D	D	D	D	D
23	F	F	F	F	F	F
24	F	F	F	F	F	F
25	P	P	P	P	P	P
26	S	S	S	S	S	S
27	NoCon	V	I	I	V	I

30	R	R	R	R	R	R	
31	L	L	L	L	L	L	
32	A	A	A	A	A	A	
33	D	D	D	D	D	D	
34	NoCon	A	E	E	E	E	
35	NoCon	D	G	G	G	G	
36	L	L	L	L	L	L	
37	N	N	N	N	N	N	
38	R	R	R	R	R	R	
39	R	R	R	R	R	R	
40	V	V	V	V	V	V	
41	A	A	A	A	A	A	
42	E	E	E	E	E	E	
43	D	D	D	D	D	D	
44	L	L	L	L	L	L	
45	N	N	N	N	N	N	
46	L	L	L	L	L	L	
47	G	G	G	G	G	G	
48	N	N	N	N	N	N	
49	L	L	L	L	L	L	
50	N	N	N	N	N	N	
51	V	V	V	V	V	V	
52	S	S	S	S	S	S	
53	I	I	I	I	I	I	
54	P	P	P	P	P	P	
55	W	W	W	W	W	W	
56	T	T	T	T	T	T	
57	H	H	H	H	H	H	
58	K	K	K	K	K	K	
59	V	V	V	V	V	V	
60	G	G	G	G	G	G	
61	N	N	N	N	N	N	
62	F	F	F	F	F	F	
63	T	T	T	T	T	T	
64	G	G	G	G	G	G	
65	L	L	L	L	L	L	
66	Y	Y	Y	Y	Y	Y	
67	S	S	S	S	S	S	
68	S	S	S	S	S	S	
69	T	T	T	T	T	T	
70	V	V	V	V	V	V	
71	P	P	P	P	P	P	
72	NoCon	I	C	V	V	V	
73	F	F	F	F	F	F	
74	N	N	N	N	N	N	
75	P	P	P	P	P	P	
76	NoCon	E	K	E	H	E	
77	W	W	W	W	W	W	

28	R	R	R	R	R	R
29	D	D	D	D	D	D
30	L	L	L	L	L	L
31	L	L	L	L	L	L
32	D	D	D	D	D	D
33	T	T	T	T	T	T
34	A	A	A	A	A	A
35	S	S	S	S	S	S
36	A	A	A	A	A	A
37	L	L	L	L	L	L
38	Y	Y	Y	Y	Y	Y
39	R	R	R	R	R	R
40	E	E	E	E	E	E
41	A	A	A	A	A	A
42	L	L	L	L	L	L
43	E	E	E	E	E	E
44	S	S	S	S	S	S
45	P	P	P	P	P	P
46	E	E	E	E	E	E
47	H	H	H	H	H	H
48	C	C	C	C	C	C
49	S	S	S	S	S	S
50	P	P	P	P	P	P
51	H	H	H	H	H	H
52	H	H	H	H	H	H
53	T	T	T	T	T	T
54	A	A	A	A	A	A
55	L	L	L	L	L	L
56	R	R	R	R	R	R
57	Q	Q	Q	Q	Q	Q
58	A	A	A	A	A	A
59	I	I	I	I	I	I
60	L	L	L	L	L	L
61	C	C	C	C	C	C
62	W	W	W	W	W	W
63	G	G	G	G	G	G
64	E	E	E	E	E	E
65	L	L	L	L	L	L
66	M	M	M	M	M	M
67	NoCon	T	N	N	T	T
68	L	L	L	L	L	L
69	A	A	A	A	A	A
70	T	T	T	T	T	T
71	W	W	W	W	W	W
72	V	V	V	V	V	V
73	G	G	G	G	G	G
74	NoCon	N	S	S	G	S
75	N	N	N	N	N	N

78	NoCon	Q	Q	Q	K	Q	
79	T	T	T	T	T	T	
80	P	P	P	P	P	P	
81	S	S	S	S	S	S	
82	F	F	F	F	F	F	
83	P	P	P	P	P	P	
84	NoCon	K	D	H	N	K	
85	I	I	I	I	I	I	
86	H	H	H	H	H	H	
87	L	L	L	L	L	L	
88	NoCon	Q	Q	Q	H	Q	
89	NoCon	E	E	E	Q	E	
90	D	D	D	D	D	D	
91	I	I	I	I	I	I	
92	NoCon	I	V	I	I	V	
93	NoCon	N	D	N	K	D	
94	NoCon	R	R	R	K	R	
95	C	C	C	C	C	C	
96	NoCon	Q	K	Q	E	K	
97	Q	Q	Q	Q	Q	Q	
98	NoCon	F	F	Y	F	F	
99	V	V	V	V	V	V	
100	G	G	G	G	G	G	
101	P	P	P	P	P	P	
102	L	L	L	L	L	L	
103	T	T	T	T	T	T	
104	V	V	V	V	V	V	
105	N	N	N	N	N	N	
106	E	E	E	E	E	E	
107	NoCon	K	N	K	K	K	
108	R	R	R	R	R	R	
109	R	R	R	R	R	R	
110	L	L	L	L	L	L	
111	NoCon	K	K	K	Q	K	
112	L	L	L	L	L	L	
113	I	I	I	I	I	I	
114	M	M	M	M	M	M	
115	P	P	P	P	P	P	
116	A	A	A	A	A	A	
117	R	R	R	R	R	R	
118	F	F	F	F	F	F	
119	Y	Y	Y	Y	Y	Y	
120	P	P	P	P	P	P	
121	NoCon	T	N	N	N	N	
122	NoCon	H	V	L	V	V	
123	T	T	T	T	T	T	
124	K	K	K	K	K	K	
125	Y	Y	Y	Y	Y	Y	

76	L	L	L	L	L	L
77	E	E	E	E	E	E
78	D	D	D	D	D	D
79	P	P	P	P	P	P
80	A	A	A	A	A	A
81	S	S	S	S	S	S
82	R	R	R	R	R	R
83	NoCon	D	E	E	D	E
84	L	L	L	L	L	L
85	V	V	V	V	V	V
86	V	V	V	V	V	V
87	NoCon	N	S	S	S	S
88	Y	Y	Y	Y	Y	Y
89	V	V	V	V	V	V
90	N	N	N	N	N	N
91	NoCon	T	V	V	T	V
92	N	N	N	N	N	N
93	M	M	M	M	M	M
94	G	G	G	G	G	G
95	L	L	L	L	L	L
96	K	K	K	K	K	K
97	NoCon	I	I	I	F	I
98	R	R	R	R	R	R
99	Q	Q	Q	Q	Q	Q
100	L	L	L	L	L	L
101	L	L	L	L	L	L
102	W	W	W	W	W	W
103	F	F	F	F	F	F
104	H	H	H	H	H	H
105	I	I	I	I	I	I
106	S	S	S	S	S	S
107	C	C	C	C	C	C
108	L	L	L	L	L	L
109	T	T	T	T	T	T
110	F	F	F	F	F	F
111	G	G	G	G	G	G
112	R	R	R	R	R	R
113	E	E	E	E	E	E
114	T	T	T	T	T	T
115	V	V	V	V	V	V
116	NoCon	L	L	L	I	I
117	E	E	E	E	E	E
118	Y	Y	Y	Y	Y	Y
119	L	L	L	L	L	L
120	V	V	V	V	V	V
121	S	S	S	S	S	S
122	F	F	F	F	F	F
123	G	G	G	G	G	G

126	L	L	L	L	L	L	
127	P	P	P	P	P	P	
128	L	L	L	L	L	L	
129	D	D	D	D	D	D	
130	K	K	K	K	K	K	
131	G	G	G	G	G	G	
132	I	I	I	I	I	I	
133	K	K	K	K	K	K	
134	P	P	P	P	P	P	
135	Y	Y	Y	Y	Y	Y	
136	Y	Y	Y	Y	Y	Y	
137	P	P	P	P	P	P	
138	NoCon	D	E	E	E	E	
139	NoCon	Q	H	H	H	H	
140	NoCon	V	V	A	L	A	
141	V	V	V	V	V	V	
142	N	N	N	N	N	N	
143	H	H	H	H	H	H	
144	Y	Y	Y	Y	Y	Y	
145	F	F	F	F	F	F	
146	NoCon	Q	Q	K	Q	Q	
147	T	T	T	T	T	T	
148	R	R	R	R	R	R	
149	H	H	H	H	H	H	
150	Y	Y	Y	Y	Y	Y	
151	L	L	L	L	L	L	
152	H	H	H	H	H	H	
153	T	T	T	T	T	T	
154	L	L	L	L	L	L	
155	W	W	W	W	W	W	
156	K	K	K	K	K	K	
157	A	A	A	A	A	A	
158	G	G	G	G	G	G	
159	I	I	I	I	I	I	
160	L	L	L	L	L	L	
161	Y	Y	Y	Y	Y	Y	
162	K	K	K	K	K	K	
163	R	R	R	R	R	R	
164	E	E	E	E	E	E	
165	NoCon	T	S	T	T	T	
166	T	T	T	T	T	T	
167	NoCon	R	R	R	H	R	
168	S	S	S	S	S	S	
169	A	A	A	A	A	A	
170	S	S	S	S	S	S	
171	F	F	F	F	F	F	
172	C	C	C	C	C	C	
173	G	G	G	G	G	G	

124	V	V	V	V	V	V
125	W	W	W	W	W	W
126	I	I	I	I	I	I
127	R	R	R	R	R	R
128	T	T	T	T	T	T
129	P	P	P	P	P	P
130	P	P	P	P	P	P
131	A	A	A	A	A	A
132	Y	Y	Y	Y	Y	Y
133	R	R	R	R	R	R
134	P	P	P	P	P	P
135	P	P	P	P	P	P
136	N	N	N	N	N	N
137	A	A	A	A	A	A
138	P	P	P	P	P	P
139	I	I	I	I	I	I
140	L	L	L	L	L	L
141	S	S	S	S	S	S
142	T	T	T	T	T	T
143	L	L	L	L	L	L
144	P	P	P	P	P	P
145	E	E	E	E	E	E
146	T	T	T	T	T	T
147	T	T	T	T	T	T
148	V	V	V	V	V	V
149	V	V	V	V	V	V
			-	-		
			-	-		
150	R	R	R	R	R	R
151	R	R	R	R	R	R
				-		
				-		
152	R	R	R	R	R	R
					-	
					-	
153	NoCon	D	G	G	G	D
154	R	R	R	R	R	R
		-				
155	NoCon	G	S	S	S	G
156	NoCon	R	P	P	P	R
					-	
	NoCon	S				S
	NoCon	P				P
157	R	R	R	R	R	R
158	R	R	R	R	R	R
159	R	R	R	R	R	R
160	T	T	T	T	T	T
161	P	P	P	P	P	P

174	S	S	S	S	S	S	
175	P	P	P	P	P	P	
176	Y	Y	Y	Y	Y	Y	
177	S	S	S	S	S	S	
178	W	W	W	W	W	W	
179	E	E	E	E	E	E	
180	Q	Q	Q	Q	Q	Q	
181	NoCon	E	D	E	E	E	
182	L	L	L	L	L	L	
183	Q	Q	Q	Q	Q	Q	
184	H	H	H	H	H	H	
185	G	G	G	G	G	G	
186	NoCon	R	R	R	A		
187	NoCon	L	L	L	-		
188	NoCon	V	V	V	-		
189	NoCon	I	F	F	-		
190	NoCon	K	Q	Q	-		
191	NoCon	T	T	T	E		
192	S	S	S	S	S		
193	NoCon	Q	K	T	F		
194	NoCon	R	R	R			
195	H	H	H	H	H		
196	NoCon	G	G	G	Q		
197	NoCon	D	D	D			
198	NoCon	E	K	E			
199	NoCon	S	S	S			
200	NoCon	F	F	F			
201	NoCon	C	C	C			
202	NoCon	S	P	S			
203	Q	Q	Q	Q	Q		
204	NoCon	P	S	S	S		
205	NoCon	S	P	S	S		
206	G	G	G	G	G		
207	I	I	I	I	I		
208	L	L	L	L	L		
209	NoCon	S	P	S	S		
210	R	R	R	R	R		
211	NoCon	S	S	S	P		
212	NoCon	S	S	P	P		
213	V	V	V	V	V		
214	G	G	G	G	G		
215	NoCon	P	P	P	S		
216	NoCon	C	C	C	S		
217	NoCon	I	I	V	L		
218	NoCon	R	Q	R	Q		
219	S	S	S	S	S		
220	NoCon	Q	Q	Q	K		
221	NoCon	L	L	L	H		

162	S	S	S	S	S	S
163	P	P	P	P	P	P
164	R	R	R	R	R	R
165	R	R	R	R	R	R
166	R	R	R	R	R	R
167	R	R	R	R	R	R
168	S	S	S	S	S	S
169	Q	Q	Q	Q	Q	Q
170	S	S	S	S	S	S
171	P	P	P	P	P	P
172	R	R	R	R	R	R
173	R	R	R	R	R	R
174	R	R	R	R	R	R
175	R	R	R	R	R	R
176	S	S	S	S	S	S
177	Q	Q	Q	Q	Q	Q
178	S	S	S	S	S	S
179	R	R	R	R	R	R
180	E	E	E	E	E	E
			-			
181	S	S	S	S	S	S
					-	
182	Q	Q	Q	Q	Q	Q
183	C	C	C	C	C	C
184	-	-	-	-	-	

222	NoCon	K	R	K	R		
223	NoCon	Q	K	Q	K		
224	S	S	S	S	S		
225	R	R	R	R	R		
226	L	L	L	L	L		
227	G	G	G	G	G		
228	NoCon	L	P	L	L		
229	Q	Q	Q	Q	Q		
230	NoCon	P	P	P	S		
231	NoCon	H	A	Q	Q		
232	Q	Q	Q	Q	Q		
233	G	G	G	G	G		
234	NoCon	P	Q	S	H		
235	L	L	L	L	L		
236	A	A	A	A	A		
237	NoCon	S	G	R	R		
238	NoCon	S	R	G	R		
239	NoCon	Q	Q	K	Q		
240	NoCon	P	Q	S	Q		
241	G	G	G	G	G		
242	NoCon	R	G	R	R		
243	S	S	S	S	S		
244	NoCon	G	G	G	W		
245	S	S	S	S	S		
246	I	I	I	I	I		
247	R	R	R	R	R		
248	A	A	A	A	A		
249	NoCon	R	R	R	G		
250	NoCon	A	V	V	I		
251	H	H	H	H	H		
252	P	P	P	P	P		
253	NoCon	S	S	T	T		
254	NoCon	T	P	T	A		
255	NoCon	R	W	R	R		
256	NoCon	R	G	R	R		
257	NoCon	Y	T	S	P		
258	NoCon	F	V	F	F		
259	G	G	G	G	G		
260	V	V	V	V	V		
261	E	E	E	E	E		
262	P	P	P	P	P		
263	S	S	S	S	S		
264	G	G	G	G	G		
265	S	S	S	S	S		
266	G	G	G	G	G		
267	NoCon	H	P	H	H		
268	NoCon	I	T	I	T		
269	NoCon	D	H	D	T		

270	NoCon	H	N	N	N		
271	NoCon	S	C	S	L		
272	NoCon	V	A	A	A		
273	NoCon	N	S	S	S		
274	NoCon	N	S	S	K		
275	NoCon	S	S	T	S		
276	NoCon	S	S	S	A		
277	S	S	S	S	S		
278	C	C	C	C	C		
279	L	L	L	L	L		
280	NoCon	H	H	H	Y		
281	Q	Q	Q	Q	Q		
282	S	S	S	S	S		
283	NoCon	A	A	A	P		
284	V	V	V	V	V		
285	R	R	R	R	R		
286	K	K	K	K	K		
287	NoCon	A	A	T	A		
288	A	A	A	A	A		
289	Y	Y	Y	Y	Y		
290	NoCon	S	S	S	P		
291	NoCon	H	L	H	A		
292	NoCon	L	I	L	V		
293	S	S	S	S	S		
294	T	T	T	T	T		
295	NoCon	S	S	S	F		
296	NoCon	K	K	K	E		
297	NoCon	R	G	R	K		
298	NoCon	Q	H	Q	H		
299	S	S	S	S	S		
300	S	S	S	S	S		
301	S	S	S	S	S		
302	G	G	G	G	G		
303	H	H	H	H	H		
304	A	A	A	A	A		
305	V	V	V	V	V		
306	E	E	E	E	E		
307	NoCon	F	L	L	L		
308	H	H	H	H	H		
309	NoCon	C	H	N	N		
310	NoCon	L	F	I	L		
311	P	P	P	P	P		
312	P	P	P	P	P		
313	NoCon	S	N	S	N		
314	S	S	S	S	S		
315	NoCon	A	S	A	A		
316	NoCon	G	R	R	R		
317	S	S	S	S	S		

318	Q	Q	Q	Q	Q			
319	S	S	S	S	S			
320	NoCon	Q	Q	E	E			
321	NoCon	G	G	G	R			
322	NoCon	S	P	P	P			
323	NoCon	V	V	I	V			
324	NoCon	F	L	L	F			
325	NoCon	S	S	S	P	S		
326	C	C	C	C	C	C		
327	W	W	W	W	W	W		
328	W	W	W	W	W	W		
329	L	L	L	L	L	L		
330	Q	Q	Q	Q	Q	Q		
331	F	F	F	F	F	F		
332	R	R	R	R	R	R		
333	N	N	N	N	N	N		
334	S	S	S	S	S	S		
335	NoCon	K	E	K	K	K		
336	P	P	P	P	P	P		
337	C	C	C	C	C	C		
338	S	S	S	S	S	S		
339	NoCon	E	E	D	D	E		
340	Y	Y	Y	Y	Y	Y		
341	C	C	C	C	C	C		
342	L	L	L	L	L	L		
343	NoCon	S	C	T	S	T		
344	H	H	H	H	H	H		
345	NoCon	L	I	I	I	L		
346	V	V	V	V	V	V		
347	N	N	N	N	N	N		
348	L	L	L	L	L	L		
349	NoCon	R	I	L	L	L		
350	E	E	E	E	E	E		
351	D	D	D	D	D	D		
352	W	W	W	W	W	W		
353	G	G	G	G	G	G		
354	P	P	P	P	P	P		
355	C	C	C	C	C	C		
356	NoCon	D	T	T	A	D		
357	E	E	E	E	E	E		
358	H	H	H	H	H	H		
359	G	G	G	G	G	G		
360	E	E	E	E	E	E		
361	H	H	H	H	H	H		
362	NoCon	H	R	N	H	H		
363	I	I	I	I	I	I		
364	R	R	R	R	R	R		
365	NoCon	I	T	I	I	I		

366	P	P	P	P	P	P	
367	R	R	R	R	R	R	
368	T	T	T	T	T	T	
369	P	P	P	P	P	P	
370	A	A	A	A	A	A	
371	R	R	R	R	R	R	
372	V	V	V	V	V	V	
373	T	T	T	T	T	T	
374	G	G	G	G	G	G	
375	G	G	G	G	G	G	
376	V	V	V	V	V	V	
377	F	F	F	F	F	F	
378	L	L	L	L	L	L	
379	V	V	V	V	V	V	
380	D	D	D	D	D	D	
381	K	K	K	K	K	K	
382	N	N	N	N	N	N	
383	P	P	P	P	P	P	
384	H	H	H	H	H	H	
385	N	N	N	N	N	N	
386	T	T	T	T	T	T	
387	NoCon	A	T	T	A	A	
388	E	E	E	E	E	E	
389	S	S	S	S	S	S	
390	R	R	R	R	R	R	
391	L	L	L	L	L	L	
392	V	V	V	V	V	V	
393	V	V	V	V	V	V	
394	D	D	D	D	D	D	
395	F	F	F	F	F	F	
396	S	S	S	S	S	S	
397	Q	Q	Q	Q	Q	Q	
398	F	F	F	F	F	F	
399	S	S	S	S	S	S	
400	R	R	R	R	R	R	
401	G	G	G	G	G	G	
402	NoCon	I	N	S	N	I	
403	NoCon	T	T	T	Y	T	
404	NoCon	R	R	H	R	R	
405	V	V	V	V	V	V	
406	S	S	S	S	S	S	
407	W	W	W	W	W	W	
408	P	P	P	P	P	P	
409	K	K	K	K	K	K	
410	F	F	F	F	F	F	
411	A	A	A	A	A	A	
412	V	V	V	V	V	V	
413	P	P	P	P	P	P	

414	N	N	N	N	N	N	
415	L	L	L	L	L	L	
416	Q	Q	Q	Q	Q	Q	
417	S	S	S	S	S	S	
418	L	L	L	L	L	L	
419	T	T	T	T	T	T	
420	N	N	N	N	N	N	
421	L	L	L	L	L	L	
422	L	L	L	L	L	L	
423	S	S	S	S	S	S	
424	S	S	S	S	S	S	
425	N	N	N	N	N	N	
426	L	L	L	L	L	L	
427	S	S	S	S	S	S	
428	W	W	W	W	W	W	
429	L	L	L	L	L	L	
430	S	S	S	S	S	S	
431	L	L	L	L	L	L	
432	D	D	D	D	D	D	
433	V	V	V	V	V	V	
434	S	S	S	S	S	S	
435	A	A	A	A	A	A	
436	A	A	A	A	A	A	
437	F	F	F	F	F	F	
438	Y	Y	Y	Y	Y	Y	
439	H	H	H	H	H	H	
440	NoCon	I	L	I	L	I	
441	P	P	P	P	P	P	
442	L	L	L	L	L	L	
443	H	H	H	H	H	H	
444	P	P	P	P	P	P	
445	A	A	A	A	A	A	
446	A	A	A	A	A	A	
447	M	M	M	M	M	M	
448	P	P	P	P	P	P	
449	H	H	H	H	H		H
450	L	L	L	L	L		L
451	L	L	L	L	L		L
452	NoCon	I	V	V	V		V
453	G	G	G	G	G		G
454	S	S	S	S	S		S
455	S	S	S	S	S		S
456	G	G	G	G	G		G
457	L	L	L	L	L		L
458	NoCon	S	S	P	S		S
459	R	R	R	R	R		R
460	Y	Y	Y	Y	Y		Y
461	V	V	V	V	V		V

462	A	A	A	A	A		A
463	R	R	R	R	R		R
464	L	L	L	L	L		L
465	S	S	S	S	S		S
466	S	S	S	S	S		S
467	NoCon	N	N	T	N		N
468	S	S	S	S	S		S
469	NoCon	R	R	R	R		R
470	NoCon	I	I	-	I		I
471	NoCon	N	I	-	F		I
472	NoCon	N	N	N	N		N
473	NoCon	N	N	-			H
474	NoCon	Q	Q	I			Q
475	NoCon	Y	H	-	H		H
476	NoCon	G	R	-	-		G
477	NoCon	T	T	N	-		T
478	NoCon	M	M	Y	-		M
479	Q	Q	Q	Q	Q		Q
480	NoCon	N	N	D	N		N
481	L	L	L	L	L		L
482	H	H	H	H	H		H
483	NoCon	D	N	D	D		D
484	S	S	S	S	S		S
485	C	C	C	C	C		C
486	S	S	S	S	S		S
487	R	R	R	R	R		R
488	NoCon	Q	N	N	N		N
489	L	L	L	L	L		L
490	Y	Y	Y	Y	Y		Y
491	V	V	V	V	V		V
492	S	S	S	S	S		S
493	L	L	L	L	L		L
494	NoCon	M	M	L	L		L
495	L	L	L	L	L		L
496	L	L	L	L	L		L
497	Y	Y	Y	Y	Y		Y
498	NoCon	K	K	K	Q		K
499	T	T	T	T	T		T
500	NoCon	Y	Y	F	F		F
501	G	G	G	G	G		G
502	NoCon	W	R	R	R		R
503	K	K	K	K	K		K
504	L	L	L	L	L		L
505	H	H	H	H	H		H
506	L	L	L	L	L		L
507	Y	Y	Y	Y	Y		Y
508	S	S	S	S	S		S
509	H	H	H	H	H		H

510	P	P	P	P	P		P
511	I	I	I	I	I		I
512	NoCon	V	I	I	I		I
513	L	L	L	L	L		L
514	G	G	G	G	G		
515	F	F	F	F	F		
516	R	R	R	R	R		
517	K	K	K	K	K		
518	I	I	I	I	I		
519	P	P	P	P	P		
520	M	M	M	M	M		
521	G	G	G	G	G		
522	V	V	V	V	V		
523	G	G	G	G	G		
524	L	L	L	L	L		
525	S	S	S	S	S		
526	P	P	P	P	P		
527	F	F	F	F	F		
528	L	L	L	L	L		
529	L	L	L	L	L		
530	A	A	A	A	A		
531	Q	Q	Q	Q	Q		
532	F	F	F	F	F		
533	T	T	T	T	T		
534	S	S	S	S	S		
535	A	A	A	A	A		
536	I	I	I	I	I		
537	C	C	C	C	C		
538	S	S	S	S	S		
539	V	V	V	V	V		
540	V	V	V	V	V		
541	R	R	R	R	R		
542	R	R	R	R	R		
543	A	A	A	A	A		
544	F	F	F	F	F		
545	P	P	P	P	P		
546	H	H	H	H	H		
547	C	C	C	C	C		
548	L	L	L	L	L		
549	A	A	A	A	A		
550	F	F	F	F	F		
551	S	S	S	S	S		
552	Y	Y	Y	Y	Y		
553	M	M	M	M	M		
554	D	D	D	D	D		
555	D	D	D	D	D		
556	V	V	V	V	V		
557	V	V	V	V	V		

558	L	L	L	L	L		
559	G	G	G	G	G		
560	A	A	A	A	A		
561	K	K	K	K	K		
562	S	S	S	S	S		
563	V	V	V	V	V		
564	Q	Q	Q	Q	Q		
565	H	H	H	H	H		
566	NoCon	R	L	L	L		
567	E	E	E	E	E		
568	S	S	S	S	S		
569	L	L	L	L	L		
570	NoCon	Y	Y	F	F		
571	NoCon	T	A	T	T		
572	NoCon	A	A	S	A		
573	NoCon	V	V	I	V		
574	T	T	T	T	T		
575	N	N	N	N	N		
576	F	F	F	F	F		
577	L	L	L	L	L		
578	L	L	L	L	L		
579	S	S	S	S	S		
580	L	L	L	L	L		
581	G	G	G	G	G		
582	I	I	I	I	I		
583	H	H	H	H	H		
584	L	L	L	L	L		
585	N	N	N	N	N		
586	P	P	P	P	P		
587	NoCon	N	H	N	N		
588	K	K	K	K	K		K
589	T	T	T	T	T		T
590	K	K	K	K	K		K
591	R	R	R	R	R		R
592	W	W	W	W	W		W
593	G	G	G	G	G		G
594	Y	Y	Y	Y	Y		Y
595	S	S	S	S	S		S
596	L	L	L	L	L		L
597	NoCon	N	N	N	H		N
598	F	F	F	F	F		F
599	M	M	M	M	M		M
600	G	G	G	G	G		G
601	Y	Y	Y	Y	Y		Y
602	NoCon	I	V	V	V		V
603	I	I	I	I	I		I
604	G	G	G	G	G		G
605	NoCon	S	S	S	C		S

606	NoCon	W	W	W	Y		W
607	G	G	G	G	G		G
608	NoCon	T	T	T	S		S
609	L	L	L	L	L		L
610	P	P	P	P	P		P
611	Q	Q	Q	Q	Q		Q
612	NoCon	D	E	E	D		D
613	H	H	H	H	H		H
614	I	I	I	I	I		I
615	NoCon	V	V	V	I		I
616	NoCon	Q	Q	L	Q		Q
617	K	K	K	K	K		K
618	I	I	I	I	I		I
619	K	K	K	K	K		K
620	NoCon	H	M	Q	E		E
621	C	C	C	C	C		C
622	F	F	F	F	F		F
623	R	R	R	R	R		R
624	K	K	K	K	K		K
625	L	L	L	L	L		L
626	P	P	P	P	P		P
627	V	V	V	V	V		V
628	N	N	N	N	N		N
629	R	R	R	R	R		R
630	P	P	P	P	P		P
631	I	I	I	I	I		I
632	D	D	D	D	D		D
633	W	W	W	W	W		W
634	K	K	K	K	K		K
635	V	V	V	V	V		V
636	C	C	C	C	C		C
637	Q	Q	Q	Q	Q		Q
638	R	R	R	R	R		R
639	I	I	I	I	I		I
640	V	V	V	V	V		V
641	G	G	G	G	G		G
642	L	L	L	L	L		L
643	L	L	L	L	L		L
644	G	G	G	G	G		G
645	F	F	F	F	F		F
646	A	A	A	A	A		A
647	A	A	A	A	A		A
648	P	P	P	P	P		P
649	F	F	F	F	F		F
650	T	T	T	T	T		T
651	Q	Q	Q	Q	Q		Q
652	C	C	C	C	C		C
653	G	G	G	G	G		G

654	Y	Y	Y	Y	Y		Y
655	P	P	P	P	P		P
656	A	A	A	A	A		A
657	L	L	L	L	L		L
658	M	M	M	M	M		M
659	P	P	P	P	P		P
660	L	L	L	L	L		L
661	Y	Y	Y	Y	Y		Y
662	A	A	A	A	A		A
663	C	C	C	C	C		C
664	I	I	I	I	I		I
665	Q	Q	Q	Q	Q		Q
666	NoCon	A	A	S	S		S
667	K	K	K	K	K		K
668	Q	Q	Q	Q	Q		Q
669	A	A	A	A	A		A
670	F	F	F	F	F		F
671	T	T	T	T	T		T
672	F	F	F	F	F		F
673	S	S	S	S	S		S
674	P	P	P	P	P		P
675	T	T	T	T	T		T
676	Y	Y	Y	Y	Y		Y
677	K	K	K	K	K		K
678	A	A	A	A	A		A
679	F	F	F	F	F		F
680	L	L	L	L	L		L
681	NoCon	S	S	C	C		S
682	K	K	K	K	K		K
683	Q	Q	Q	Q	Q		Q
684	Y	Y	Y	Y	Y		Y
685	NoCon	M	L	L	L		L
686	N	N	N	N	N		N
687	L	L	L	L	L		L
688	Y	Y	Y	Y	Y		Y
689	P	P	P	P	P		P
690	V	V	V	V	V		V
691	A	A	A	A	A		A
692	R	R	R	R	R		R
693	Q	Q	Q	Q	Q		Q
694	R	R	R	R	R		R
695	NoCon	P	P	S	P		P
696	G	G	G	G	G		G
697	L	L	L	L	L		L
698	C	C	C	C	C		C
699	Q	Q	Q	Q	Q		Q
700	V	V	V	V	V		V
701	F	F	F	F	F		F

702	A	A	A	A	A		A
703	D	D	D	D	D		D
704	A	A	A	A	A		A
705	T	T	T	T	T		T
706	P	P	P	P	P		P
707	T	T	T	T	T		T
708	G	G	G	G	G		G
709	W	W	W	W	W		W
710	G	G	G	G	G		G
711	L	L	L	L	L		L
712	NoCon	A	A	A	V		A
713	NoCon	I	I	I	M		M
714	G	G	G	G	G		G
715	H	H	H	H	H		H
716	NoCon	Q	Q	R	Q		Q
717	R	R	R	R	R		R
718	M	M	M	M	M		M
719	R	R	R	R	R		R
720	G	G	G	G	G		G
721	T	T	T	T	T		T
722	F	F	F	F	F		F
723	NoCon	V	V	V	L		V
724	NoCon	A	S	A	A		A
725	P	P	P	P	P		P
726	L	L	L	L	L		L
727	P	P	P	P	P		P
728	I	I	I	I	I		I
729	H	H	H	H	H		H
730	T	T	T	T	T		T
731	A	A	A	A	A		A
732	E	E	E	E	E		E
733	L	L	L	L	L		L
734	L	L	L	L	L		L
735	A	A	A	A	A		A
736	A	A	A	A	A		A
737	C	C	C	C	C		C
738	F	F	F	F	F		F
739	A	A	A	A	A		A
740	R	R	R	R	R		R
741	S	S	S	S	S		S
742	R	R	R	R	R		R
743	S	S	S	S	S		S
744	G	G	G	G	G		G
745	A	A	A	A	A		A
746	NoCon	K	K	K	N		K
747	NoCon	L	L	L	I		I
748	NoCon	I	I	I	L		L
749	G	G	G	G	G		G

750	T	T	T	T	T		T
751	D	D	D	D	D		D
752	N	N	N	N	N		N
753	S	S	S	S	S		S
754	V	V	V	V	V		V
755	V	V	V	V	V		V
756	L	L	L	L	L		L
757	S	S	S	S	S		S
758	R	R	R	R	R		R
759	K	K	K	K	K		K
760	Y	Y	Y	Y	Y		Y
761	T	T	T	T	T		T
762	S	S	S	S	S		S
763	F	F	F	F	F		F
764	P	P	P	P	P		P
765	W	W	W	W	W		W
766	L	L	L	L	L		L
767	L	L	L	L	L		L
768	G	G	G	G	G		G
769	C	C	C	C	C		C
770	NoCon	T	A	A	A		A
771	A	A	A	A	A		A
772	N	N	N	N	N		N
773	W	W	W	W	W		W
774	I	I	I	I	I		I
775	L	L	L	L	L		L
776	R	R	R	R	R		R
777	G	G	G	G	G		G
778	T	T	T	T	T		T
779	S	S	S	S	S		S
780	F	F	F	F	F		F
781	V	V	V	V	V		V
782	Y	Y	Y	Y	Y		Y
783	V	V	V	V	V		V
784	P	P	P	P	P		P
785	S	S	S	S	S		S
786	A	A	A	A	A		A
787	L	L	L	L	L		L
788	N	N	N	N	N		N
789	P	P	P	P	P		P
790	A	A	A	A	A		A
791	D	D	D	D	D		D
792	D	D	D	D	D		D
793	P	P	P	P	P		P
794	S	S	S	S	S		S
795	R	R	R	R	R		R
796	G	G	G	G	G		G
797	R	R	R	R	R		R

798	L	L	L	L	L		L
799	G	G	G	G	G		G
800	L	L	L	L	L		L
801	NoCon	S	Y	Y	S		S
802	R	R	R	R	R		R
803	P	P	P	P	P		P
804	L	L	L	L	L		L
805	L	L	L	L	L		L
806	NoCon	R	R	H	R		R
807	L	L	L	L	L		L
808	NoCon	P	L	P	P		P
809	NoCon	F	Y	F	F		F
810	NoCon	Q	R	R	R		R
811	P	P	P	P	P		P
812	T	T	T	T	T		T
813	T	T	T	T	T		T
814	G	G	G	G	G		G
815	R	R	R	R	R		R
816	T	T	T	T	T		T
817	S	S	S	S	S		S
818	L	L	L	L	L		L
819	Y	Y	Y	Y	Y		Y
820	A	A	A	A	A		A
821	NoCon	V	D	V	D		V
822	S	S	S	S	S		S
823	P	P	P	P	P		P
824	S	S	S	S	S		S
825	V	V	V	V	V		V
826	P	P	P	P	P		
827	S	S	S	S	S		
828	H	H	H	H	H		
829	L	L	L	L	L		
830	P	P	P	P	P		
831	NoCon	V	D	D	D		
832	R	R	R	R	R		
833	V	V	V	V	V		
834	H	H	H	H	H		
835	F	F	F	F	F		
836	A	A	A	A	A		
837	S	S	S	S	S		
838	P	P	P	P	P		
839	L	L	L	L	L		
840	H	H	H	H	H		
841	V	V	V	V	V		
842	A	A	A	A	A		
843	W	W	W	W	W		
844	R	R	R	R	R		
845	P	P	P	P	P		

846	P	P	P	P	P		

https://www.hiv.lanl.gov/cgi-bin/ENTROPY/entropy_main.cgi
