# Supplementary material for: Hepatitis B virus polymerase-specific T cell epitopes shift in a mouse model of chronic infection
Source: Virol J. 2021 Dec 7;18:242. doi: 10.1186/s12985-021-01712-y (PMC8650432; doi:10.1186/s12985-021-01712-y)
Supplement: Supplementary file 2 — Additional file 2. Table S2. Consensus polymerase and core sequences [file 12985_2021_1712_MOESM2_ESM.pdf]

Additional Information Table 2. Peptide matrices

The table shows which peptides were present in the different peptide pools that were tested to identify those that elicited T cell responses in vaccinated mice.

Polymerase								
Pools	A	B	C	D	E	F	G	H
I	1	2	3	4	5	6	7	8
J	9	10	11	12	13	14	15	16
K	17	18	19	20	21	22	23	24
L	25	26	27	28	29	30	31	32
M	33	34	35	36	37	38	39	40
N	41	42	43	44	45	46	47	48
O	49	50	51	52	53	54	55	56
P	57	58	59*					

Core						
Pools	A	B	C	D	E	F
G	1	2	3	4	5	6
H	7	8	9	10	11	12
I	13	14	15	16	17	18
J	19	20	21	22	23	24
K	25	26	27	28	29	30
L	31	32	33	34	35	
