# Supplementary material for: Hepatitis B virus polymerase-specific T cell epitopes shift in a mouse model of chronic infection
Source: Virol J. 2021 Dec 7;18:242. doi: 10.1186/s12985-021-01712-y (PMC8650432; doi:10.1186/s12985-021-01712-y)
Supplement: Supplementary file 3 — Additional file 3. Figure S1. Expression of the gD fusion proteins by the vaccines. The graphs show results of Western blot for CHO-CAR cells infected with 103vp/cells of the AdC6-gDPolC and AdC6-gDPolN vectors or HEK293 cells infected with the same doses of the AdC6-gDCore, AdC7-gDPolC, AdC7-gDPolN or AdC7-gDCore vectors. An AdC vector expressing gp140 of HIV-1 was used as a negative control. An AdC vector expressing a gD-HPV oncoprotein fusion protein was used as a positive control. The blots were probed with an antibody to gD, stripped, and then tested with an antibody to ß-actin as a loading control (not shown) [file 12985_2021_1712_MOESM3_ESM.pdf]

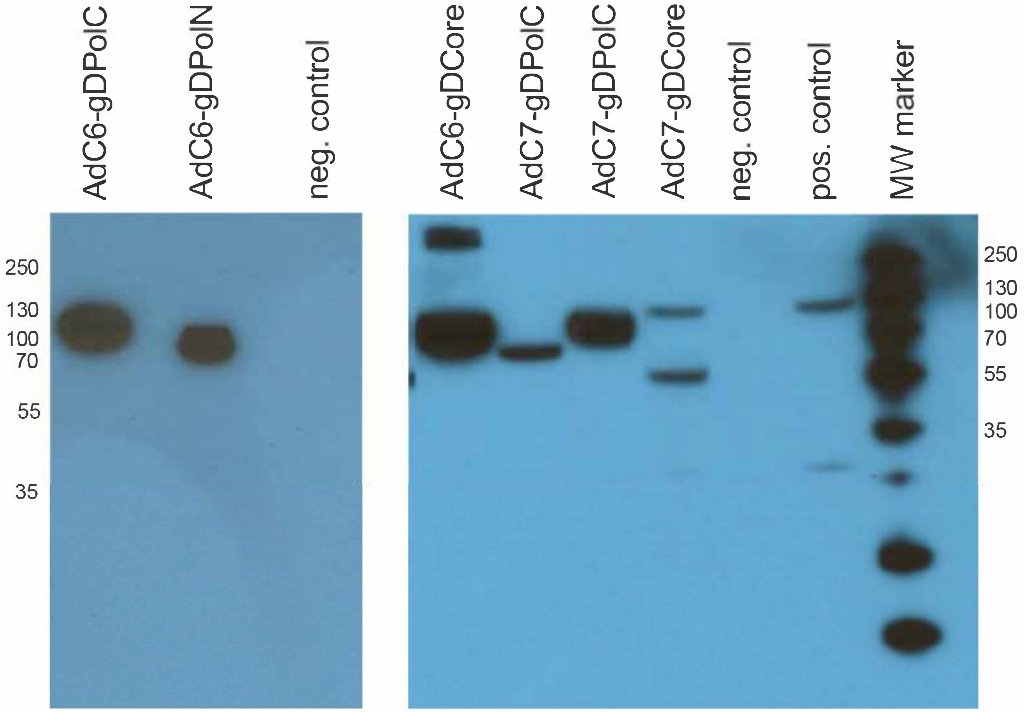

Expression of the gD fusion proteins by the vaccines.
The figures show results of Western blot for CHO-CAR cells infected with 10^3vp/cells of the AdC6-gDPolC and AdC6-gDPolN vector or HEK293 cells infected with the same doses of the AdC6-gDCore, AdC7-gDPolC, AdC7-gDPolN or AdC7-gDCore vectors. An AdC vector expressing gp140 of HIV-1 was used as a negative control. An AdC vector expressing a gD-HBV oncoprotein fusion protein was used as a positive control. The blots were probed with an antibody to gD, stripped, and then tested with an antibody to ß-actin as a loading control (not shown).
